# Assessing visibility at highway-rail grade crossings using light detection and ranging (LiDAR) technology

**DOI:** 10.1016/j.heliyon.2024.e40347

**Published:** 2024-11-13

**Authors:** Mohsen Naghdi, Pasi Lautala, Abdolmajid Erfani

**Affiliations:** Department of Civil, Environmental, and Geospatial Engineering, Michigan Technological University, 1400 Townsend Drive, Houghton, MI, 49931, USA

**Keywords:** Highway-rail grade crossings, Sightlines, Geospatial analysis, LiDAR data

## Abstract

In 2022, 2034 incidents occurred at highway-rail grade crossings (HRGCs) in the United States, posing significant risks such as fatalities, injuries, and property damage. These incidents underscore the need for effective prevention and mitigation strategies. With over 212,000 public and private HRGCs nationwide, safety monitoring is challenging, as traditional inspections primarily rely on manual assessments. Obstructed sightlines at HRGCs further increase safety risks by limiting road users' ability to see approaching trains. Although previous studies have addressed behavioral and safety issues, the literature currently lacks quantitative data analysis of sightlines at HRGCs. This study aims to address this gap by utilizing remote sensing techniques to identify and quantify sightline visibility. We studied 12 HRGCs using geospatial analysis in ArcGIS Pro through Viewshed and Observer Points analysis, integrating United States Geological Survey (USGS) Light Detection and Ranging (LiDAR) data with United States Federal Railroad Administration (FRA) crossing reports. Our findings indicate that sightline issues in the case studies reviewed are primarily linked to traffic control devices, environmental factors, and geometric conditions. Additionally, the results suggest that USGS LiDAR data and geospatial analysis offer potentially rapid and cost-effective methods for identifying sightline safety issues at HRGCs.

## Highlights

• This study uses high-resolution Light Detection and Ranging data to evaluate sightlines at highway-rail grade crossings.This study uses high-resolution Light Detection and Ranging data to evaluate sightlines at highway-rail grade crossings.
• It provides a framework to quantify sightlines at highway-rail grade crossings.
• The effectiveness of the remote sensing approach is compared with the more conventional surveying methods.
• Our proposed approach offers the potential for quick and practical enhancements to current inspection practices.

## 1 Introduction

A highway-rail grade crossing (HRGC) is a location within the transportation network where a road intersects with train tracks at the same elevation [1]. In 2022, 2034 incidents occurred at HRGCs across the United States, according to the Federal Railroad Administration (FRA) [2]. Evaluating the hazard conditions at HRGCs is essential due to the potentially severe consequences, including fatalities, injuries, and property damage. Limited visibility at HRGCs poses a significant safety hazard by obstructing the view of approaching trains. Various factors contribute to restricted visibility, such as seasonal and continuous vegetation, small crossing angles, nearby structures, adverse weather conditions like fog or snowfall, and mobile objects. When visibility is restricted, road users (referred to interchangeably as observers or drivers in this study) may struggle to accurately judge the distance and speed of approaching trains. According to the FRA, there are over 212,000 HRGCs across the country [3]. Manually inspecting such a vast number of crossings to assess visibility is both challenging and time-consuming. Regular inspections are crucial, as conditions at these crossings can change over time due to factors such as environmental conditions, infrastructure wear and tear, changes in traffic patterns, and infrastructure development or deterioration. Additionally, modifications made to improve safety or accommodate increased traffic volumes may impact risk levels. For example, vegetation growth near crossings can obstruct visibility, or changes in road configurations could alter the risk associated with a crossing.

One effective strategy to address resource constraints in manual inspections is the adoption of technologies like Light Detection and Ranging (LiDAR). LiDAR is a remote sensing technology that accurately measures distances and creates detailed three-dimensional (3D) models of environments. It operates by emitting light pulses toward a target and calculating the time it takes for the reflections to return. These reflections are recorded as numerous individual points, forming a LiDAR point cloud (LPC), which collectively represents the 3D positions of various objects on the Earth's surface. LiDAR offers unique advantages over traditional technologies by delivering 3D data that conventional imaging sensors cannot capture. This approach also reduces costs by minimizing the need for extensive travel and manual inspections, although it requires its own data collection process [4]. Traditional techniques are often time-consuming, especially for large-scale projects requiring high precision. Moreover, LiDAR improves efficiency and safety by eliminating the need for field measurements and reducing risks associated with highway and train traffic [5]. Previous studies have explored the use of LiDAR data in safety assessments at HRGCs. For instance, Wang et al. [6] employed LiDAR data to develop a 3D surface model for assessing HRGC roughness, introducing a method that uses 3D sensing and imaging technology to evaluate surface quality. Similarly, Khattak et al. [5] evaluated the feasibility of using LiDAR data to assess humped HRGCs for vehicles with low ground clearance, with field validation confirming that LiDAR accurately identified potential blockage points.

**Fig. 1:**
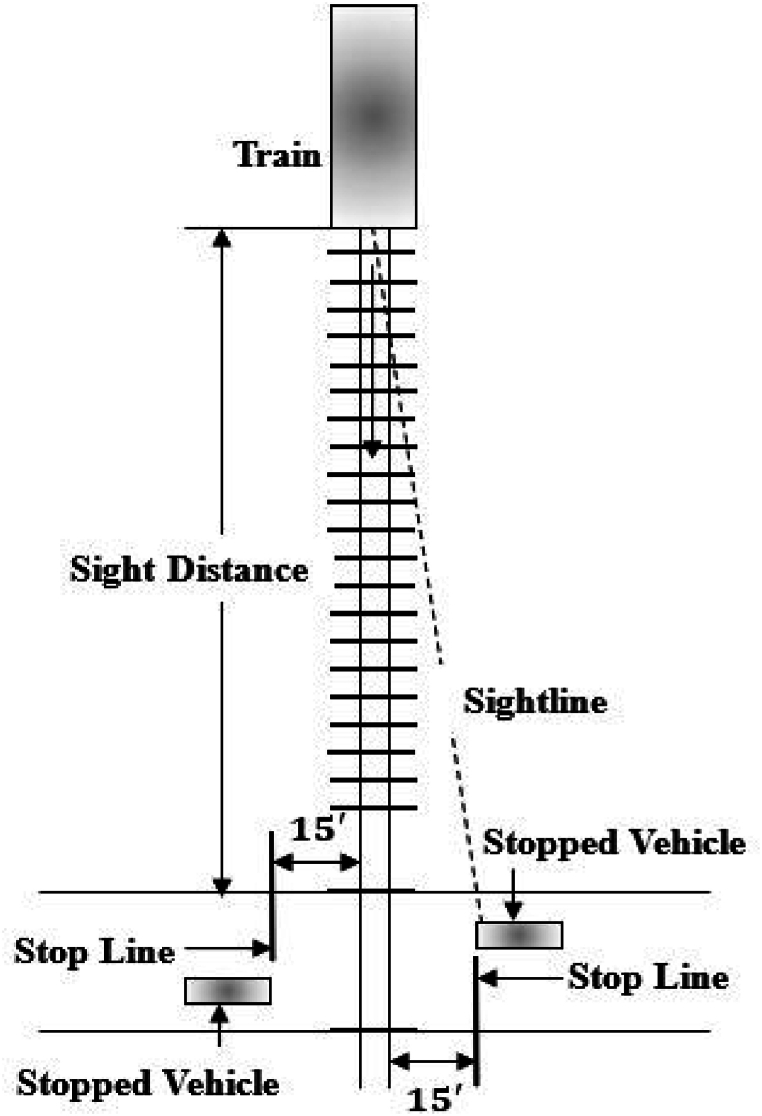
Sight Distance for Stopped Vehicle adapted from [7].

Sightline assessment is essential in evaluating visibility at HRGCs, as supported by FRA requirements and elaborated upon by Ogden and Cooper [1]. A sightline refers to the unobstructed sightline from the road user to an approaching train and involves measuring three key sight distances, including approach sight distance, corner sight distance, and clearing sight distance. Approach sight distance is the distance from the road user to the crossing at which they first become aware of it. Corner sight distance is the distance at which a moving vehicle can detect an approaching train while clearing sight distance is the distance that must be visible along the tracks for a road user in a stopped vehicle to safely clear the crossing (Fig. 1). These distances form the basis for the distance triangle, defined by the train's distance from the crossing ($d_{T}$), the minimum safe sight distance for the vehicle (whether moving or stopped) along the highway ($d_{H}$), and the sightline itself. This triangle represents the area that must be visible to ensure safe passage. It is required that vehicles should come to a complete stop at the stop line when a stop sign is present, with the stop line positioned 15 feet (ft) from the nearest rail. Fig. 1 illustrates the sight distance required for a vehicle at a complete stop.

In cases where advance warning or stop signs are absent, it is critical to evaluate the sightline for a moving vehicle approaching the crossing, examining different combinations of vehicle and train speeds to ensure that road users have adequate visibility. The sight distances required for various combinations of vehicle and train speeds are provided in Table 1.

**Table 1:** Sight distances for combinations of highway vehicle and train speeds [7].

Train Speed (MPH)	Departure from Stop	Moving Vehicle							
		Vehicle Speed (MPH)							
–	0	10	20	34	40	50	60	70	80
**Distance Along Railroad from Crossing, d**_**T**_**(ft)**									
10	255	155	110	102	102	106	112	119	127
20	509	310	220	203	205	213	225	239	254
30	794	465	331	305	307	319	337	358	381
40	1019	619	441	407	409	426	450	478	508
50	1273	774	551	509	511	532	562	597	635
60	1528	929	661	610	614	639	675	717	763
70	1783	1084	771	712	716	745	787	836	890
80	2037	1239	882	814	818	852	899	956	1017
90	2292	1394	992	915	920	958	1012	1075	1144
**Distance Along Highway from Crossing, d**_**H**_**(ft)**									
–	–	69	135	220	324	447	589	751	931

Numerous studies have been conducted to identify different potential hazards at HRGCs [8,9]. These studies aim to enhance safety measures and reduce the risk of accidents at these crossings [[10], [11], [12]]. A majority of prior research endeavors can be categorized into three primary domains, including driver behavior [[13], [14], [15], [16]], safety evaluation [[17], [18], [19], [20], [21], [22], [23]], and operational assessment [[24], [25], [26]]. By analyzing factors such as characteristics of the road user and vehicle, crossing features, environmental conditions, warning devices, traffic volume, and train frequency, researchers seek to gain a comprehensive understanding of the risks involved.

However, research on quantifying sightlines at HRGCs has received limited attention. Some studies have focused on evaluating sight distances as a safety measure. For example, Fitzpatrick et al. [27] assessed sight distance requirements for large trucks at HRGCs, revealing potential deficiencies in current criteria for moving trucks on highways while indicating adequacy for stopped vehicles along the tracks. This finding underscores the need to revisit and possibly adjust sight triangle values to ensure the safety of truck drivers at grade crossings. Khattak and Shamayleh [28] introduced a method for assessing highway sight distances using LiDAR and Geographic Information Systems (GIS). Their visual and line-of-sight analysis identified potential safety issues, which were validated during field visits, highlighting the effectiveness of LiDAR and GIS in highway safety evaluations. Recently, the Crossing-i System, an automated, drone-based grade crossing assessment tool developed by the Michigan Technological University Research Institute [29], has evaluated the risk of vehicles getting stuck on tracks due to vertical approach grades. This system also assesses sightline visibility using viewshed and Observer Points (VOP) analyses on ArcGIS Pro [30] and identifies key assets, such as gates, lights, and signage at HRGCs. Using high-resolution images captured by unmanned aerial systems, the Crossing-i system processes this data into 3D models for safety analysis.

This study contributes to the body of knowledge in sightline analysis at HRGCs by developing a framework that utilizes publicly available high-resolution United States Geological Survey (USGS) LiDAR data [31] and geospatial analysis. The framework has been applied to 12 HRGCs in the southwestern region of Duluth, Minnesota (MN). We specifically evaluated the visibility for road users when a train is approaching the HRGCs by quantifying sightlines. The selected case studies were based on various factors, including location diversity, variations in sightline conditions identified by the Crossing-i system, different geometric configurations (such as intersection angles and terrain), train speeds, and the presence of control devices like stop signs. Cases analyzed by the Crossing-i system were also included to compare and validate our findings. The LiDAR data was processed and transformed into raster datasets, facilitating the visualization and analysis of the HRGC environment. By applying VOP analyses within ArcGIS Pro [29], we identified areas with restricted visibility, which indicated potential safety concerns where sightlines were obstructed. This remote sensing framework not only identifies areas with restricted visibility but also measures and scores sightlines, providing a basis for targeted interventions to enhance safety at HRGCs. The primary contribution of this study is demonstrating that LiDAR data along Federal FRA crossing inventory data, and geospatial analysis can effectively help to quantify and measure sightline visibility at HRGCs. The insights gained enable interventions, including strategic vegetation management, timely maintenance, and immediate actions regarding control devices when necessary.

## 2 Methodology

**Fig. 2:**
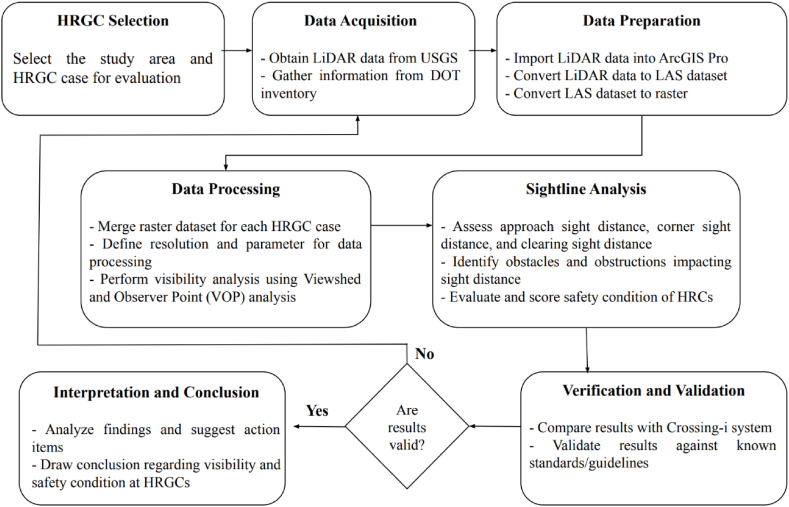
Methodology flowchart.

To examine the visibility conditions surrounding HRGCs, this study presents a practical framework for evaluating safety based on visibility levels. The proposed framework begins with the data collection for selected HRGCs, followed by the gathering of relevant geospatial data and design attributes associated with these crossings. This process includes the extraction, preparation, and processing of LiDAR data, along with Viewshed and observer Point analyses to evaluate sightlines and visibility areas. Once the visibility data has been processed, HRGCs are scored based on their average visibility levels. This scoring system provides a quantitative measure of safety related to visibility, which is critical for understanding the potential risks at these crossings. The final step in this framework involves validating the findings by comparing them with field inspection methods, such as the Crossing-i system. Fig. 2 illustrates a flowchart outlining the sequential steps of the safety assessment framework proposed in this study, providing a visual representation of the methodology and the interconnections between each stage of the evaluation process.

### 2.1 Data acquisition

**Fig. 3:**
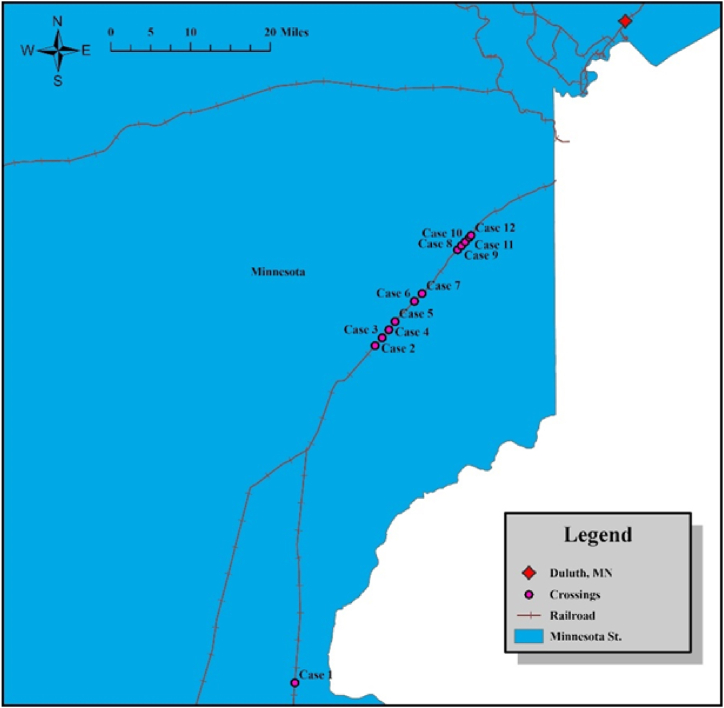
Study area mapping.

We evaluated visibility at 12 HRGC case studies selected from southwest Duluth, MN. The selection process considered several factors, including geographic diversity, different geometric configurations (such as intersection angles and terrain), varying train speeds, and the presence of safety control devices. Additionally, we considered variations in sightline conditions identified by the Crossing-i system. Fig. 3 displays the distribution of these case studies as a part of the MN state railroad network.

The HRGCs selected for this study reflect a variety of factors, including urban and rural locations, sightline conditions from clear to obstructed, diverse geometric layouts, varying train speeds, and different safety controls like stop signs. To validate the results, we also included cases previously examined by the Crossing-i system. For each crossing, we gathered data using the United States Department of Transportation (U.S. DOT) Crossing Inventory data, a standardized document used by railroads and state DOTs to report HRGC details to the FRA. This data provides information such as the crossing's name, geographic coordinates, train speed, traffic control devices, and physical characteristics like the smallest crossing angle. The data required for our analysis based on the crossing data and Table 1 is summarized in Table 2.

**Table 2:** HRGCs data Overview

Case	Stop Sign	Train Speed (mph)	Vehicle Speed (mph)	$d_{t}$ (ft), derived from Table 1	$d_{H}$ (ft), derived from Table 1
**1**	Yes	20	0	509	15
**2**	Yes	50	0	1273	15
**3**	Yes	50	0	1273	15
**4**	Yes	50	0	1273	15
**5**	No	50	15	662.5	102
**6**	No	50	30	521	196
**7**	No	50	15	662.5	102
**8**	Yes	50	0	1273	15
**9**	Yes	50	0	1273	15
**10**	Yes	50	0	1273	15
**11**	Yes	50	0	1273	15
**12**	Yes	50	0	1273	15

**Fig. 4:**
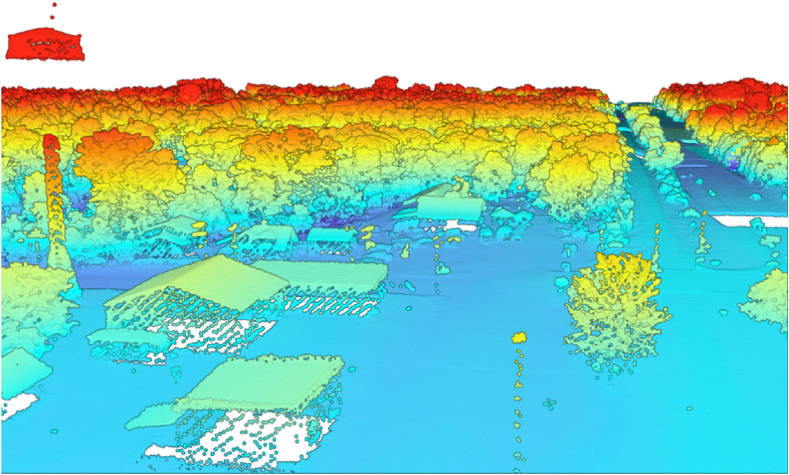
3D visualization of LiDAR point cloud data used in the study.

Additionally, we utilized USGS LPCs for precise terrain data and elevation details. LiDAR equipment is frequently mounted on airborne platforms such as airplanes, helicopters, or drones. It employs a laser scanner, a Global Positioning System (GPS), and an Inertial Navigation System (INS) to generate high-resolution ground elevation models. The system measures the distance between the sensor and the ground by emitting rapid pulses of light and calculating the time it takes for the pulses to reflect back. This data allowed us to create detailed 3D models of the topography and surrounding features for each crossing. The dataset used in this study consists of processed Classified LAS files formatted into 1000 m by 1000 m (approximately 3280.84 ft by 3280.84 ft) tiles, partially covering areas in Minnesota and Wisconsin. Data were collected in spring 2019 under favorable conditions (no snow and normal river levels). The USGS offers LiDAR data through the National Map, which provides access to a wide range of geospatial datasets. We employed the National Map Download Application (TNM Download v2.0) as the primary data source to select and download LiDAR data corresponding to targeted HRGCs. Fig. 4 showcases a 3D visualization of the LPC data for a portion of the surveyed area.

By merging high-resolution terrain data with U.S. DOT Crossing inventory data, using geographical coordinates, we successfully developed precise models of the HRGC environments. The combination of LiDAR data, crossing inventory information, and traffic control characteristics enabled us to perform a thorough evaluation of each crossing, accounting for both physical and operational variables that contribute to crossing visibility.

### 2.2 Data preparation

The raw LiDAR data, stored in LAZ format, was imported into the software for processing. To ensure accurate spatial alignment, an appropriate coordinate system was selected based on the study area's geographic extent. Once the data was imported and organized, it was converted from its original LAS format to raster. This conversion was crucial, as raster data provides a continuous surface representation ideal for visibility analysis. The raster resolution was set to 1 m, with each pixel representing a one-square-meter area, enabling precise elevation details of the surrounding terrain.

### 2.3 Data processing

**Fig. 5:**
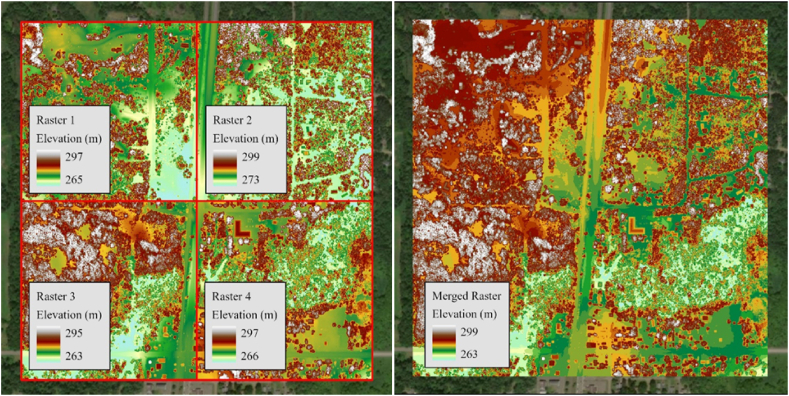
Four raster (left image) Merged to a new raster dataset (right image).

ArcGIS Pro, a widely used GIS software offered by Environmental Systems Research Institute, Inc. (ESRI), was used for data processing in this study. To cover the full area needed for the analysis of each crossing, multiple raster datasets were combined into a continuous surface. This mosaic process ensured that all necessary terrain data was included in the visibility model, regardless of whether it originated from different LiDAR datasets. Fig. 5 illustrates this process where the left side shows four individual raster datasets prior to merging, while the right side presents the seamless raster after merging the datasets.

Once the raster datasets were prepared and combined, the locations of each crossing, road users, and trains in both directions were pinpointed using graphical map layers, which were then converted into feature layers. These features provided the geographic context necessary for visibility analysis, enabling the study to determine which parts of the landscape would be visible to a driver at each HRGC. Various tools, including Viewshed, Observer Points, and Line of Sight, are available within ArcGIS Pro for visibility analysis. The Viewshed tool generates a raster indicating how many times each location is visible from specified observer points. The Observer Points tool provides binary information on which observation points can see each raster cell, specifying the exact observer points that have a direct line of sight to each location on the raster surface. In this study, we utilized the Viewshed tool as the primary method for assessing visibility and employed the Observer Points tool to identify which observation points could see each raster. This approach facilitated further investigation of crossings identified with limited visibility.

### 2.4 Visibility assessment

The main goal of the proposed framework is to measure sightline visibility at HRGCs to identify dangerous crossings and rank them based on their visibility scores. To evaluate the risk level at each crossing, we developed a scoring system based on the visibility of approaching trains from various directions as observed by individuals at the site. We defined five potential visibility scenarios—ranging from no visibility to full visibility—and assigned scores to each as follows.

1. No Visibility: At this level, no observer, regardless of their direction relative to the crossing, can see any approaching train from either direction. This represents the highest challenging condition, with an average visibility score of 0.
2. Limited Visibility (One Direction): In this scenario, an observer can only see one approaching train in one direction but has no sight of a train in the other direction. This limited visibility condition is assigned an average visibility score of 0.25.
3. Limited Visibility (Two Directions): Observers stationed at various points around the crossing may either each see one approaching train from a different direction, or one observer can see trains coming from both directions while the other observer has no sightline. This scenario is assigned an average visibility score of 0.5.
4. Limited Visibility (Three Directions): One observer can see an approaching train from one direction, while another observer can see approaching trains from both directions. This condition is given an average visibility score of 0.75.
5. Full Visibility: This represents the optimal visibility condition, where both observers, regardless of their position relative to the crossing, have sufficient sightlines of all approaching trains. Crossings with full visibility are assigned the highest score of 1.

These scored visibility levels offer an objective measure for assessing the safety of HRGCs, helping to prioritize interventions where visibility is poorest. Crossings that score a 0 present the highest risk and require immediate attention, while crossings scoring 1 exhibit optimal visibility and the lowest risk. This scoring system enables authorities to further investigate potential risks and make informed decisions about necessary improvements to enhance safety at critical crossings.

## 3 Results

In this section, the authors first summarize the results of the analysis. Based on these results, we then focus on two cases for each visibility scenario, including full visibility, limited visibility, and no visibility. Table 3 summarizes the results and visibility assessment carried out in this study.

**Table 3:** Visibility assessment results

Case	$d_{t}$ (ft)	Dir.	Sight Line (ft) OBS 1	Sight Line (ft) OBS 2	Visibility Score	Average Visibility Score	Visible Area (ft^2^)	Visibility Assessment
**1**	509	N	>509	>509	1	1	139,619	Visible to both drivers in both directions.
		S	>509	>509	1			
**2**	1273	N	>1273	>1273	1	1	204,784	Visible to both drivers in both directions.
		S	>1273	>1273	1			
**3**	1273	N	908	1107	0	0.5	130,362	Limited, not visible to both drivers, each in one direction.
		S	>1273	>1273	1			
**4**	1273	N	>1273	>1273	1	1	192,179	Visible to both drivers in both directions.
		S	>1273	>1273	1			
**5**	662.5	N	>1273	>1273	1	0.75	121,632	Limited, not visible to one driver in one direction.
		S	851	>1273	0.5			
**6**	521	N	361	394	0	0	34,757	Not Visible to both drivers
		S	426	36	0			
**7**	662.5	N	232	200	0	0	44,670	Not Visible to both drivers
		S	217	327	0			
**8**	1273	N	>1273	>1273	1	1	508,562	Visible to both drivers in both directions.
		S	>1273	>1273	1			
**9**	1273	N	>1273	>1273	1	1	142,891	Visible to both drivers in both directions.
		S	>1273	>1273	1			
**10**	1273	N	>1273	>1273	1	1	273,780	Visible to both drivers in both directions.
		S	>1273	>1273	1			
**11**	1273	N	116	332	0	0.5	133,709	Limited, not visible to both drivers, each in one direction.
		S	>1273	>1273	1			
**12**	1273	N	>1273	>1273	1	1	97,004	Visible to both drivers in both directions.
		S	>1273	>1273	1			

In each case, we assessed the sightlines for two observers (OBS 1 and OBS 2 in Table 3) in both directions along the train track. When the sightline matched or exceeded the required sight distance (d_T_), the framework assigned a visibility score of 1; otherwise, a score of 0 is assigned. The average of these scores appears in the "Average Visibility Score" column. Crossings with an average score of 1 indicate sufficient sight distance, while those with lower scores require further investigation. The analysis reveals a range of sightlines and visible areas, with a minimum of 34,752 square feet (ft^2^) and a maximum of 508,562 ft^2^ visible area. According to Table 3, two cases (6 and 7) fall into the no visibility scenario. Three cases (3, 5, and 11) exhibit limited visibility. The remaining seven cases (1, 2, 4, 8, 9, 10, and 12) are classified under full visibility category. In the following, we present a detailed assessment of two representative cases from each scenario. In this study, Observer 1 and Observer 2 refer to road users traveling in the northbound and southbound directions of the highway at the crossing. Similarly, Train 1 and Train 2 represent trains approaching from the north and south, respectively. For simplicity, we refer to the directions of Train 1 and Train 2 as north and south, even though their actual orientations deviate from true north and south. We set the observers' height and the target height of the train at 3.5 ft (1.08 m) and 2.0 ft (0.6 m), respectively, according to the guidelines provided by the American Association of State Highway and Transportation Officials (AASHTO) [7].

### 3.1 No visibility

Case 6 and Case 7 fall into the no visibility category. We present the results of both Viewshed and Observer Point and compare them to available open-source satellite imagery maps to gain insights into these crossings.

#### 3.1.1 Case 6

**Fig. 6:**
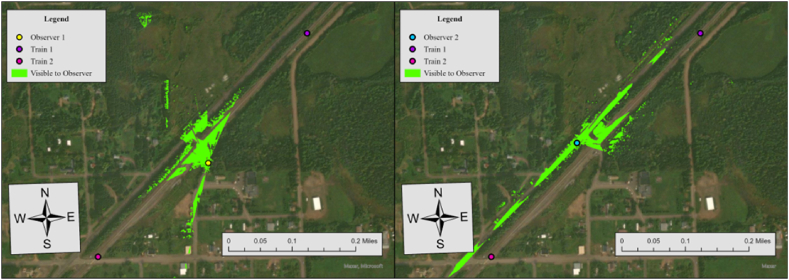
Viewshed analysis results for observer 1 (left) and observer 2 (right), case 6.

According to the crossing inventory data, there is no advance warning or stop signs at this crossing. The maximum train speed and limited highway speed are 50 miles per hour (mph) and 30 mph, respectively. Hence, the sight distance from the crossing to trains $\left(d_{T}\right)$ in either direction and the sight distance from the driver to the crossing $\left(d_{H}\right)$ were configured at 521 ft and 196 ft in our analysis based on Table 2. Fig. 6 shows the results of viewshed analyses conducted for two observers at this crossing.

**Fig. 7:**
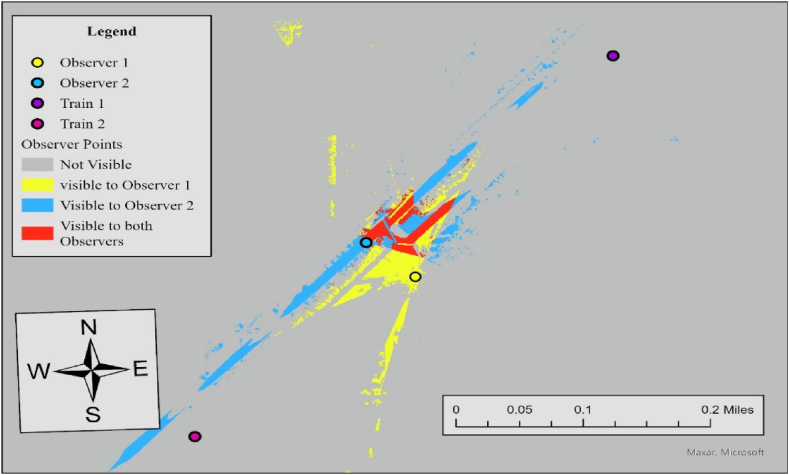
Observer points analysis results for observer 1 and observer 2, case 6.

The results of viewshed analyses provide a spatial representation of visibility conditions at each HRGC. In Case 6, the required sight distance $\left(d_{T}\right)$ is 521 ft, but the measured sightlines for both observers fall short of this requirement. For Observer 1, the sightline in the north direction is 361 ft and 394 ft in the south direction, while Observer 2's sightlines are 426 ft to the north and 36 ft to the south. Since none of the measured sightlines meet the required distance, the visibility score for both directions is 0, resulting in an average visibility score of 0. To further specify sightline conditions at this crossing, we additionally performed the Observer Points analysis. Fig. 7 illustrates the result.

The output raster shows regions that are not visible to any observer, areas visible to Observer 1, areas visible to Observer 2, and regions visible to both observers. Red regions indicate areas visible to both observers simultaneously, which is 34,757 ft^2^. Both observers lack visibility to trains approaching from either direction, consistent with the visibility assessment in Table 3. The no visibility status at this crossing is influenced by three key factors. Firstly, the lack of sufficient traffic control devices, such as advance warning or stop signs, creates a larger sight triangle compared to scenarios where vehicles stop at the stop line. Secondly, overgrown vegetation, including tall bushes and trees, obstructs the sightlines within the sight triangle. Lastly, the geometric features of the surrounding area, comprising elevation changes, slopes, and contours, play a role in further impeding visibility.

#### 3.1.2 Case 7

**Fig. 8:**
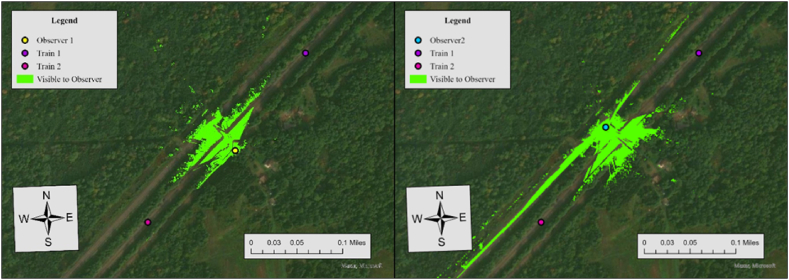
Viewshed analysis results for observer 1 (left) and observer 2 (right), case 7.

Similar to Case 6, there is no advance warning or stop signs at this crossing. The maximum train and limited highway speeds are 50 mph and 15 mph, respectively. Hence, the sight distance from the crossing to trains $\left(d_{T}\right)$ in either direction and the sight distance from the driver to the crossing $\left(d_{H}\right)$, were configured at 662.5 ft and 102 ft in our analysis based on Table 2. Fig. 8 shows the results of viewshed analyses conducted for two observers at this crossing.

**Fig. 9:**
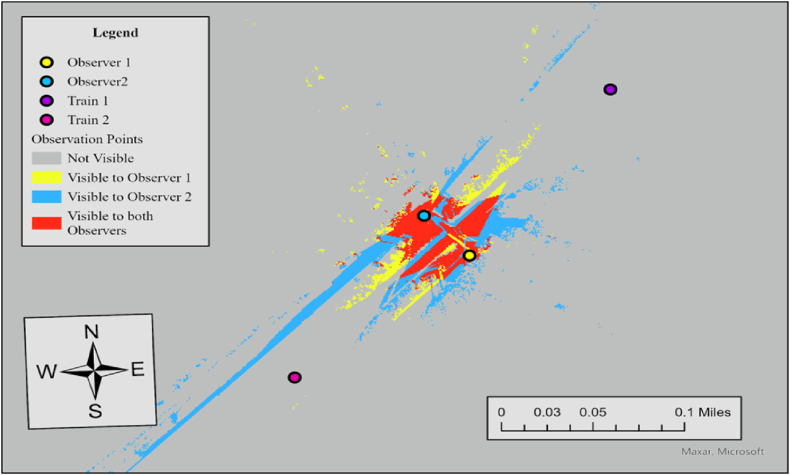
Observer points analysis results for observer 1 and observer 2, case 7.

In Case 7, the required sight distance $\left(d_{T}\right)$ is 662.5 ft, but the measured sightlines for both observers fall short of this requirement. For Observer 1, the sightline in the north direction is 232 ft and 217 ft in the south direction, while Observer 2's sightlines are 200 ft to the north and 327 ft to the south. Since none of the measured sightlines meet the required distance, the visibility score for both directions is 0, resulting in an average visibility score of 0. To further specify sightline conditions at this crossing, we additionally performed Observer Points analysis. Fig. 9 illustrates the result.

Both observers lack visibility to trains approaching from either direction, consistent with the visibility assessment in Table 3. The visible area to both observers (red area) is 44,670 ft^2^, and trains approaching in either direction are not visible to both drivers. The no visibility status at this crossing is influenced by three key factors, similar conditions at Case 6; However, a reduction in the limit highway speed from 30 mph to 15 mph resulted in overall more visibility area, compared to Case 6.

### 3.2 Limited visibility

Case 3, Case 5, and Case 11 fall into the no limited visibility category. We selected Case 3 and Case 11 for further demonstration. This section presents the findings from the viewshed and Observer Point analyses, as well as satellite imagery to enhance our understanding of these crossings.

#### 3.2.1 Case 3

**Fig. 10:**
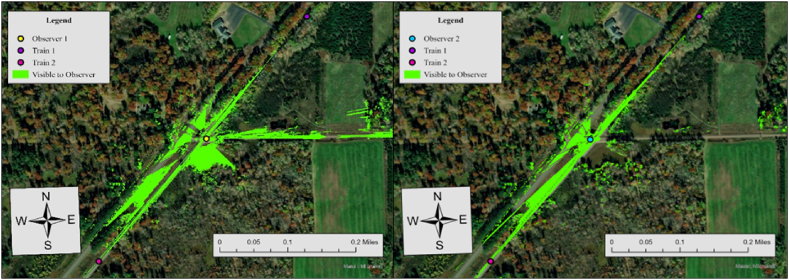
Viewshed analysis results for observer 1 (left) and observer 2 (right), case 3.

Considering that stop signs exist in this crossing, the sight distance from the crossing to trains $\left(d_{T}\right)$ were configured at 1273 ft based on Table 2. Fig. 10 shows the results of viewshed analyses conducted for two observers at this crossing.

**Fig. 11:**
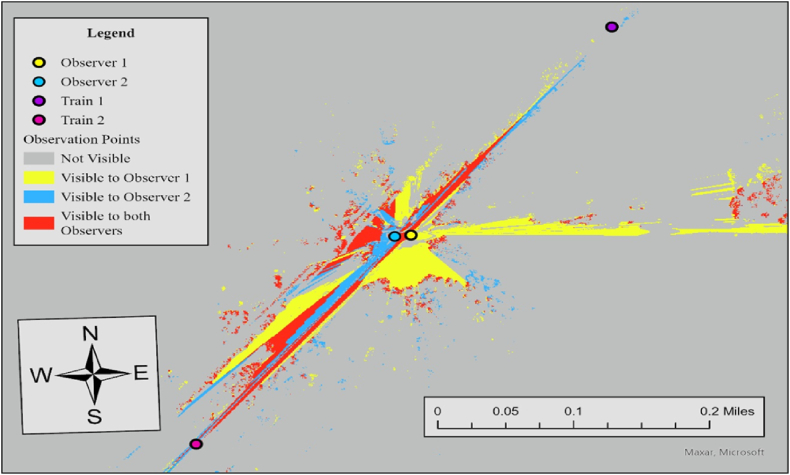
Observer points analysis results for observer 1 and observer 2, case 3.

In this case, the required sight distance $\left(d_{T}\right)$ is 1273 ft, but the measured sightlines for both observers in the north direction fall short of this requirement. For Observer 1, the sightline in the north direction is 908 ft, while Observer 2's sightline in the north direction is 1107 ft. Since one of the two measured sightlines meets the required distance, the visibility score for the north direction is 0, resulting in an average visibility score of 0.5. To further specify sightline conditions at this crossing, we additionally performed Observer Points analysis. Fig. 11 illustrates the result.

Both observers have limited visibility of trains approaching from the north, as noted in the visibility assessment in Table 3. Considering that stop signs exist at the crossing, drivers are required to stop at the stop lines, which are positioned 15 ft before the nearest rail. The visible area to both observers (red area) is 130,362 ft^2^, and trains approaching in either direction are not visible to both drivers. The limited visibility at this crossing is primarily caused by overgrown vegetation along the rail tracks, which should be removed or trimmed regularly to improve safety.

#### 3.2.2 Case 11

**Fig. 12:**
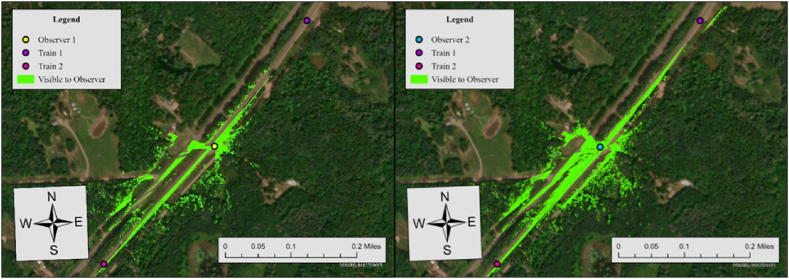
Viewshed analysis results for observer 1 (left) and observer 2 (right), case 11.

Similar to Case 3, stop signs are present at this crossing, and the sight distance to approaching trains $\left(d_{T}\right)$ is measured at 1273 ft, as shown in Table 2. Fig. 12 illustrates the results of the viewshed analysis conducted for two observers at this crossing.

**Fig. 13:**
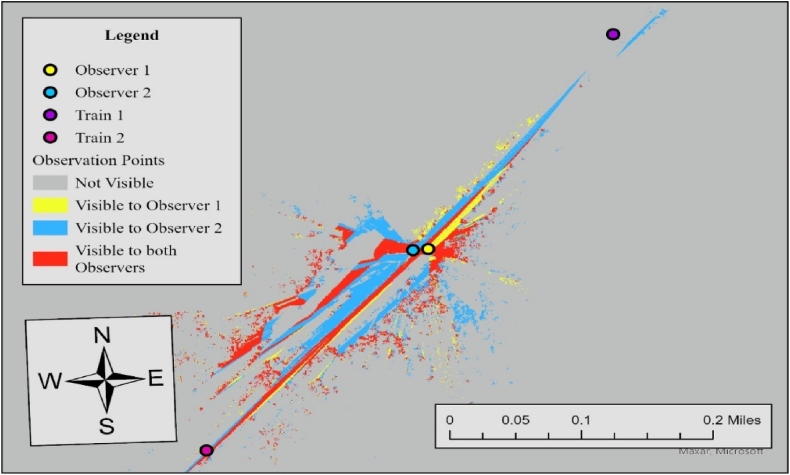
Observer points analysis results for observer 1 and observer 2, case 11.

In this case, the required sight distance $\left(d_{T}\right)$ is 1273 ft, but the sightlines for both observers in the north does not satisfy this requirement. For Observer 1, the sightline in the north direction is 116 ft, while Observer 2's sightline in the north direction is 332 ft. Considering that one of the two sightlines in each crossing meets the required distance, the average visibility score is 0.5. Fig. 13 illustrates the result of the Observer Points analysis.

Both observers have limited visibility of trains approaching from the north. With stop signs present at the crossing, drivers are required to stop at the stop line, positioned 15 ft before the nearest rail. The visible area for both observers covers 133,709 ft^2^, but trains approaching from either direction are not visible to the drivers. The limited visibility at this crossing is primarily due to a horizontal curve on the north side of the train tracks. This curve obstructs sightlines for both drivers. To improve safety, additional measures such as enhance warning signs, flashing lights, etc., should be considered to better alert road users to approaching trains.

### 3.3 Full visibility

Among the studied cases, we selected Case 1 and Case 8 for detailed assessment. Both cases have clear sight triangle areas and sufficient sightlines in both directions. Additionally, we compared the results of Case 1 with those obtained from the Crossing-i system to validate our findings.

#### 3.3.1 Case 1

**Fig. 14:**
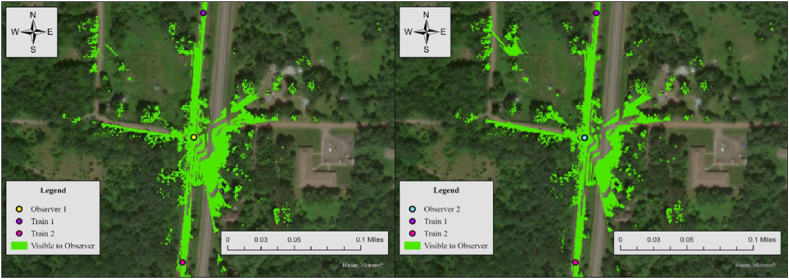
Viewshed analysis results for observer 1 (left) and observer 2 (right), case 1.

The maximum train speed of 20 mph as it is located in an urban area. Fig. 14 illustrates the results of viewshed analyses.

**Fig. 15:**
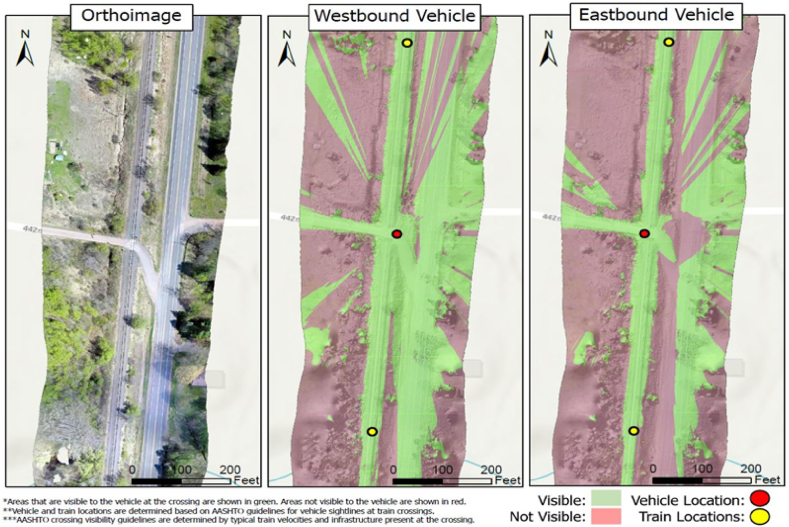
Viewshed Analysis Results obtained from the Crossing-i system, Case 1.

Both drivers have sufficient sightlines in both directions. Low train speed resulted in a required sight distance $\left(d_{T}\right)$ of 509 ft compared to our other case studies. By comparing the results from the analyses conducted by the Crossing-i system, we can derive additional insights. Fig. 15 illustrates the visibility analysis results generated by the Crossing-i system, which employs high-resolution images captured by a drone, converting them into 3D models for safety assessment.

**Fig. 16:**
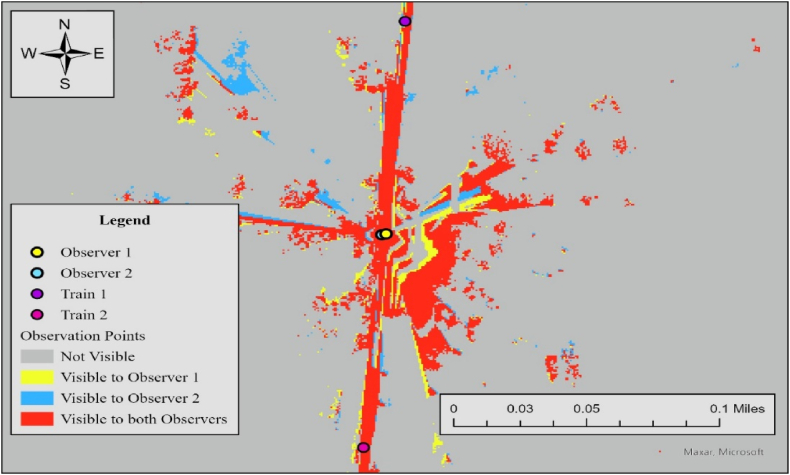
Observer points analysis results for observer 1 and observer 2, case 11.

The visible area for both observers covers 139,619 (ft^2^). Fig. 16 illustrates the results of the observer points analysis for Case 1. Since all sightlines meet the required sight distance, the average visibility score is 1.

#### 3.3.2 Case 8

**Fig. 17:**
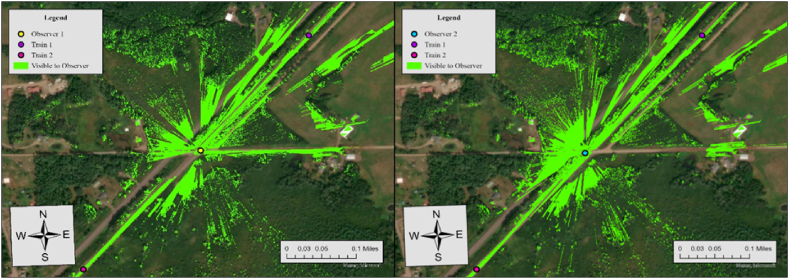
Viewshed analysis results for observer 1 (left) and observer 2 (right), case 8.

This case involves a controlled crossing with a stop sign that requires vehicles to come to a complete stop. Furthermore, because the maximum speed of incoming trains is limited to 50 mph, they are positioned 1202 ft away from the crossing. Fig. 17 illustrates the results of viewshed analyses.

**Fig. 18:**
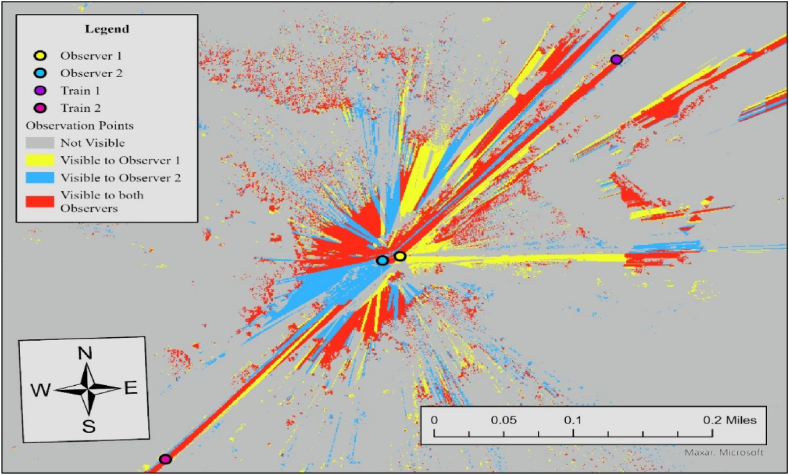
Observer points analysis results for observer 1 and observer 2, case 8.

In this case, the required sight distance $\left(d_{T}\right)$ is 1273 ft, and both drivers have sightlines more than this in both directions. Compared to Case 1, although $\left(d_{T}\right)$ in this case is about 2.5 times longer, favorable safety measures still lead to a visible crossing for both drivers. Fig. 18 shows the results of Observer Points analyses.

## 4 Discussion

While previous research has extensively explored behavioral and safety concerns at HRGCs, there is a notable gap in quantitative data focused on sightlines. Our study, successfully identified HRGCs with poor visibility, providing a valuable tool for quantifying sightlines at crossings. Our findings indicate that sightline-related safety concerns at HRGCs are primarily due to inadequate control devices, obstructive vegetation (seasonal or perennial), and challenging geometric designs. In several cases, the FRA's sight distance requirements were not met. One critical issue was the absence of advance warning or stop signs at certain HRGCs, which significantly impacted visibility. Without these control devices, drivers required longer sightlines and larger sight triangles compared to equipped crossings, as they needed more time to detect approaching trains and stop safely. In the most severe cases, up to 94 % of the sightlines were obstructed. This not only includes sightlines but also reduces the total visible area at the crossing, negatively impacting driver awareness and reaction times. In addition to control devices, vegetation overgrowth emerged as a key factor in poor visibility. At one of the studied crossings, vegetation obstructed up to 29 % of sightlines. Proactive vegetation management is crucial, as regularly scheduled maintenance could prevent many of these visibility issues. Geometric design, such as vertical or horizontal curvature of the train tracks, also plays a role in sightline obstructions and must be evaluated for further improvements. It is important to note that for calculations involving stopping sight distance and decision sight distance, a height of 2.00 feet (0.60 m) is commonly used, as outlined in the AASHTO guidelines [7]. This height represents the smallest object that typically poses a risk to drivers and corresponds to the height of automobile headlights and taillights. Using a lower object height would increase the length required for crest vertical curves without clear evidence of enhanced safety. While longer crest vertical curves might reduce crash frequency, they come with potentially significant construction costs. This choice underscores the trade-off between conservative design practices and practical implementation. In our study, this object height was chosen to balance realistic driver perception with cost-effective infrastructure design. Future research should consider this factor, as selecting a different object height could refine sight distance analysis and improve safety recommendations for HRGCs.

While USGS LiDAR data may only be updated every few years, it remains a valuable resource for long-term change detection. By comparing datasets from different years, authorities can track vegetation growth over time and predict when trimming or clearing is necessary, leading to more proactive management and fewer emergency interventions. For future research, integrating Artificial Intelligence-based techniques [[32], [33], [34]] such as object detection algorithms with remote sensing data could enable more automated and efficient sightline assessments at HRGCs. High-resolution imagery could help detect obstructions or model sightlines under different crossing conditions, facilitating better safety evaluations. Another promising direction is the use of virtual simulations in GIS or Augmented Reality to evaluate the impact of sightlines on safety perception and decision-making remotely.

## 5 Conclusion

Restricted sightlines at HRGCs significantly endanger safety by blocking views of oncoming trains, often due to factors like vegetation, sharp track-road angles, nearby structures, and adverse weather conditions. These obstructions make it difficult for road users to accurately judge the distance and speed of approaching trains, increasing safety risks. Manual inspection of HRGCs to assess sightlines is both time-consuming and costly. This study proposes a framework to evaluate the sightline and visibility scores of HRGCs by utilizing publicly available high-resolution USGS LiDAR data and geospatial analysis. 12 HRGCs were investigated using the proposed approach, revealing prevalent issues including topographical obstacles, overgrown vegetation, and malfunctioning control devices. These results underscore the importance of using LiDAR and geospatial analysis as complementary tools alongside other remote sensing techniques to effectively enhance safety at HRGCs. The method proposed in this study demonstrates promising results, particularly when compared to drone-based, high resolution image on-site investigations like the Crossing-i system. By leveraging LiDAR data, the study provides an assessment of visibility levels and identifies unsafe crossings, offering a robust basis for prioritizing safety interventions.

Overall, the findings emphasize the potential of LiDAR technology in performing visibility analysis at HRGCs. While further research and development are necessary to refine and expand the applicability of this approach, the study underscores the importance of leveraging advanced technologies to ensure the safe passage of road users and trains at HRGCs. Although the framework offers a cost-effective and straightforward method to assess the sightline conditions of a crossing, it requires a substantial amount of high-resolution data and careful selection of crossing parameters.

## CRediT authorship contribution statement

**Mohsen Naghdi:** Writing – review & editing, Writing – original draft, Visualization, Methodology, Investigation, Formal analysis, Data curation. **Pasi Lautala:** Writing – review & editing, Resources, Project administration, Methodology, Conceptualization. **Abdolmajid Erfani:** Writing – review & editing, Supervision, Project administration, Methodology.

## Declaration of competing interest

The authors declare that they have no known competing financial interests or personal relationships that could have appeared to influence the work reported in this paper.

## References

[1] Ogden B., Cooper C. (2019). https://railroads.dot.gov/gxhandbook.

[2] Federal Railroad Administration (2024). Highway/rail grade crossing incidents. https://railroads.dot.gov/safety-data/accident-and-incident-reporting/highwayrail-grade-crossing-incidents/highwayrail-grade.

[3] Federal Railroad Administration (2024). Highway-rail grade crossing and trespassing research. https://railroads.dot.gov/research-development/program-areas/highway-rail-grade-crossing/highway-rail-grade-crossing-and.

[4] Hill J.M., Graham L., Henry R.J., Cotter D., Young D.K. (2000). Wide-area topographic mapping and applications using airborne light detection and ranging (LIDAR) technology. Photogramm. Eng. Rem. Sens..

[5] Khattak A.J., Tang Z., Lee M. (2015).

[6] Wang T., Souleyrette R., Lau D., Xu P. (2014). Proceedings of the Joint Rail Conference.

[7] American Association of State Highway and Transportation Officials (AASHTO) (2018). http://www.transportation.org.

[8] Ahmed J., Robinson A., Miller E.E. (2024). Effectiveness of signs for pedestrian-railroad crossings: colors, shapes, and messaging strategies. J. Saf. Res..

[9] Keramati A., Lu P., Ren Y., Tolliver D., Ai C. (2021). Investigating the effectiveness of safety countermeasures at highway-rail at-grade crossings using a competing risk model. J. Saf. Res..

[10] Bondarabadi M.A., Rahimi H., Arefkhani H., Kashani A.T. (2023). A new approach to assess safety performance of rail regions with an emphasis on the resources and equipment of each region. Journal of Rail Transport Planning & Management.

[11] Bridgelall R., Tolliver D.D. (2024). Railroad accident analysis by machine learning and natural language processing. Journal of Rail Transport Planning & Management.

[12] Zhou X., Lu P., Zheng Z., Tolliver D., Keramati A. (2020). Accident prediction accuracy assessment for highway-rail grade crossings using random forest algorithm compared with decision tree. Reliab. Eng. Syst. Saf..

[13] Lautala P., Muhire M., Salim A., Jeon M., Nelson D., Dean A. (2017).

[14] Mounce J.M. (1981). Driver compliance with stop-sign control at low-volume intersections. Transport. Res. Rec..

[15] Richards S.H., Heathington K.W. (1988). Motorist understanding of railroad highway grade crossing traffic control devices and associated traffic laws. Transport. Res. Rec..

[16] Tung L.W., Khattak A. (2015). Distracted motor vehicle driving at highway–rail grade crossings. Transport. Res. Rec..

[17] Metaxatos P., Sriraj P.S. (2016). Pedestrian safety at rail grade crossings: focus areas for research and intervention. Urban Rail Transit.

[18] Russo B.J., James E., Erdmann T., Smaglik E.J. (2020). Pedestrian and bicyclist behavior at highway-rail grade crossings: an observational study of factors associated with violations, distraction, and crossing speeds during train crossing events. J. Transport. Saf. Secur..

[19] Moon Y.J., Coleman F.I.I.I. (2002). Dynamic dilemma zone based on driver behavior and car-following model at highway–rail intersections. Transp. Res. Part B Methodol..

[20] Haleem K., Gan A. (2015). Contributing factors of crash injury severity at public highway-railroad grade crossings in the US. J. Saf. Res..

[21] Zhao S., Khattak A. (2014). Motor vehicle drivers' injuries in train–motor vehicle crashes. Accid. Anal. Prev..

[22] Haleem K. (2016). Investigating risk factors of traffic casualties at private highway-railroad grade crossings in the United States. Accid. Anal. Prev..

[23] Keramati A., Lu P., Tolliver D., Wang X. (2020). Geometric effect analysis of highway-rail grade crossing safety performance. Accid. Anal. Prev..

[24] Chandler C., Hoel L.A. (2004). Center for Transportation Studies.

[25] Okitsu W., Louie J., Lo K. (2010). Paper Presented at the ITE Western District Annual Meeting.

[26] Beanland V., Grant E., Read G.J., Stevens N., Thomas M., Lenné M.G., Salmon P.M. (2018). Challenging conventional rural rail level crossing design: evaluating three new systems thinking-based designs in a driving simulator. Saf. Sci..

[27] Fitzpatrick K., Mason J., Glennon J. (1989). Sight distance requirements for trucks at railroad-highway grade crossings. Transport. Res. Rec..

[28] Khattak A.J., Shamayleh H. (2005). Highway safety assessment through geographic information system-based data visualization. J. Comput. Civ. Eng..

[29] Michigan Tech Research Institute (MTRI), Inc (2024). Crossing-i system. https://webserver.mtri.org/wordpress/mtri_dot_org/rail-crossing-assessment/.

[30] ESRI (2023). Using viewshed and observer points for visibility analysis. https://pro.arcgis.com/en/pro-app/latest/tool-reference/3d-analyst/using-viewshed-and-observer-points-for-visibility.htm.

[31] United States Geological Survey (2022). https://apps.nationalmap.gov/downloader/.

[32] Zhang L., Zhang L. (2022). Artificial intelligence for remote sensing data analysis: a review of challenges and opportunities. IEEE Geoscience and Remote Sensing Magazine.

[33] Erfani A., Cui Q. (2021). Natural language processing application in construction domain: an integrative review and algorithms comparison. Computing in Civil Engineering.

[34] Tang R., De Donato L., Besinović N., Flammini F., Goverde R.M., Lin Z., Wang Z. (2022). A literature review of Artificial Intelligence applications in railway systems. Transport. Res. C Emerg. Technol..

